# Study in Natural Time of Geoelectric Field and Seismicity Changes Preceding the M_w_6.8 Earthquake on 25 October 2018 in Greece

**DOI:** 10.3390/e20110882

**Published:** 2018-11-16

**Authors:** Nicholas V. Sarlis, Efthimios S. Skordas

**Affiliations:** 1 Section of Solid State Physics, Department of Physics, National and Kapodistrian University of Athens, Panepistimiopolis, Zografos, 15784 Athens, Greece; 2 Solid Earth Physics Institute, Department of Physics, National and Kapodistrian University of Athens, Panepistimiopolis, Zografos, 15784 Athens, Greece

**Keywords:** earthquakes, seismic electric signals, natural time analysis, entropy, nowcasting earthquakes

## Abstract

A strong earthquake of magnitude M${}_{w}$6.8 struck Western Greece on 25 October 2018 with an epicenter at 37.515${}^{\circ }$ N 20.564${}^{\circ }$ E. It was preceded by an anomalous geolectric signal that was recorded on 2 October 2018 at a measuring station 70 km away from the epicenter. Upon analyzing this signal in natural time, we find that it conforms to the conditions suggested for its identification as precursory Seismic Electric Signal (SES) activity. Notably, the observed lead time of 23 days lies within the range of values that has been very recently identified as being statistically significant for the precursory variations of the electric field of the Earth. Moreover, the analysis in natural time of the seismicity subsequent to the SES activity in the area candidate to suffer this strong earthquake reveals that the criticality conditions were obeyed early in the morning of 18 October 2018, i.e., almost a week before the strong earthquake occurrence, in agreement with earlier findings. Finally, when employing the recent method of nowcasting earthquakes, which is based on natural time, we find an earthquake potential score around 80%.

## 1. Introduction

**Figure 1:**
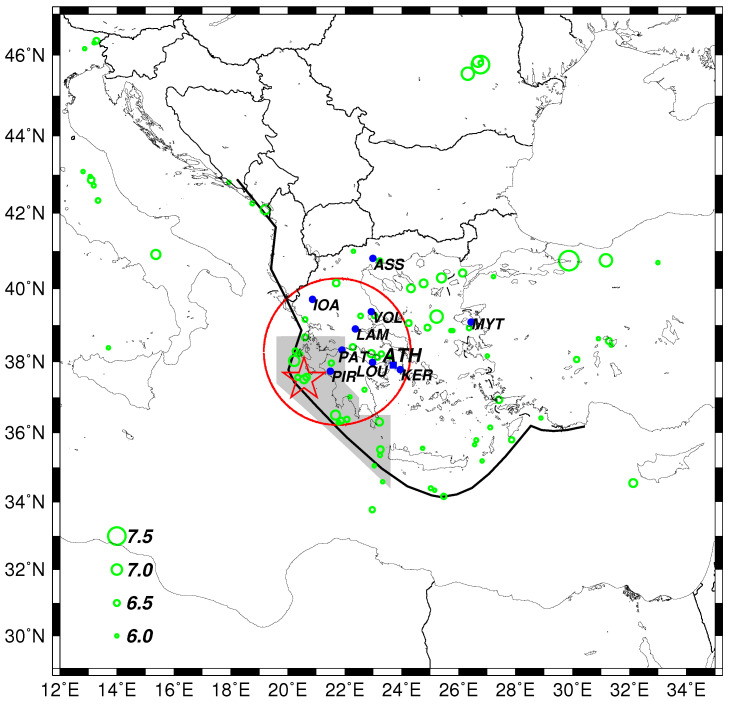
Map of the larger area $N_{29}^{47}E_{12}^{35}$ in which the EQs of magnitude greater than or equal to 6.0 are shown by the green circles. The locations of the measuring stations operating in Greece of the VAN telemetric network are shown by the blue circles. The thick black line depicts the Hellenic arc [3] while the gray shaded area the selectivity map of Pirgos (PIR) measuring station. The red star corresponds to the epicenter of the M${}_{w}$6.8 EQ on 25 October 2018 and the red circle delimits a circular region with radius R=225 km around the city of Patras.

According to the United States Geological Survey (USGS) [1], a strong earthquake (EQ) of moment magnitude M${}_{w}$6.8 occurred on 25 October 2018 10:55 p.m. Coordinated Universal Time (UTC) at an epicentral distance around 133 km SW of the city of Patras, Western Greece. Patras has a metropolitan area inhabited by a quarter of a million persons and fatal casualties have been probably avoided because, among others, at 10:23 p.m. UTC, almost half an hour before the strong EQ, a moderate EQ of magnitude M = 5.0 occurred approximately at the same area as the strong EQ [2] (see Figure 1).

**Figure 2:**
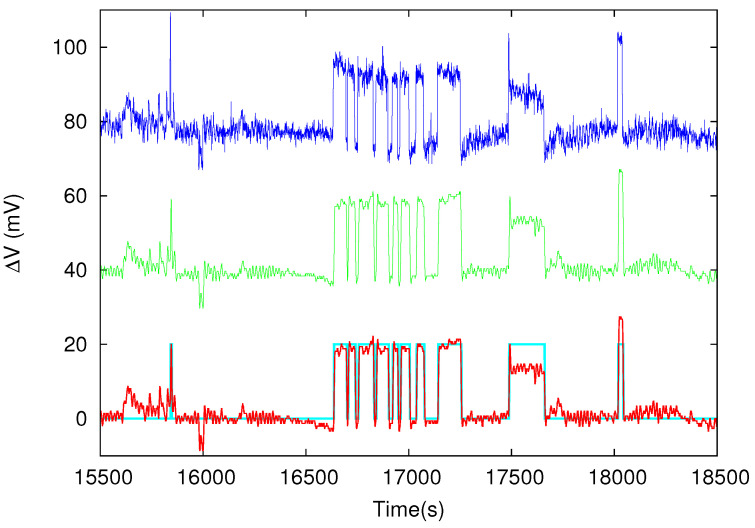
The SES activity recorded at three (almost parallel) measuring dipoles at PIR station. The time is measured in seconds since 00:00 UTC on 2 October 2018. The dichotomous representation of the SES activity is shown in the bottom channel by the cyan line.

Geoelectric field continuous monitoring is operating in Greece by the Solid Earth Physics Institute [4,5,6] at nine measuring field stations (see the blue circles in Figure 1) aiming at detecting Seismic Electric Signals (SES). SES are low frequency (≤1 Hz) variations of the electric field of the Earth that have been found to precede strong EQs in Greece [7,8,9,10,11], Japan [12,13,14], China [15,16,17,18], Mexico [19,20], and elsewhere [21]. They are emitted due to the cooperative orientation of the electric dipoles [5,22,23] (that exist anyhow due to defects [24,25] in the rocks) of the future focal area when the gradually increasing stress before the strong EQ reaches a critical value [10]. SES may appear either as single pulses or in the form of SES activities, i.e., many pulses within a relatively short time period ([9], e.g., see Figure 2). The lead time of single SES is less than or equal to 11 days, while, for SES activities, it varies from a few weeks up to 5$\frac{1}{2}$ months [6,9]. SES are recorded [9,10] at sensitive points [26] on the Earth’s surface that have been selected after long experimentation in Greece during the 1980s and 1990s that led [27,28] to the construction of the so-called VAN telemetric network (from the acronym of the scientists Varotsos, Alexopoulos and Nomicos who pioneered this research) comprising the nine measuring field stations depicted in Figure 1. Each measuring station records SES from specific EQ prone areas which constitute the so-called selectivity map of the station [3,10,29]. The gray shaded area of Figure 1 depicts the selectivity map for the Pirgos (PIR) measuring station as it resulted after the recording of SES from various epicentral areas [30]. A basic criterion for distinguishing SES from noise is that the recorded signal should [9] exhibit properties compatible with the fact that it was emitted far away from the recording station. This is usually called [9] ΔV/L criterion (where ΔV stands for the potential difference between two electrodes that constitute a measuring electric dipole and *L* for the distance between them) and has been found [31,32,33,34] to be compatible with the aforementioned SES generation model if we take into account that EQs occur in faults (where resistivity is usually orders of magnitude smaller than that of the surrounding rocks, e.g., see [5] and references therein).

The SES research has been greatly advanced after the introduction of the concept of natural time in 2001 [35,36,37]. Firstly, the criticality properties of SES activities (like the existence of long-range correlations and unique entropic properties) has been revealed by natural time analysis and hence new possibilities have been provided for the identification of SES and their distinction from man-made noise [11,38,39,40,41,42,43]. Secondly, natural time analysis allowed the introduction of an order parameter for seismicity the study of which allows the determination of the occurrence time of the strong EQ within a few days up to one week [6,30,40,44,45,46,47]. Thirdly, minima of the fluctuations of the order parameter of seismicity have been identified before all shallow EQs with M≥7.6 in Japan during the 27 year period from 1 January 1984 to 11 March 2011, the date of the M9.0 Tohoku EQ occurrence [48,49]. Finally, the interrelation of SES activities and seismicity has been further clarified because, when studying the EQ magnitude time series in Japan, it was found that the minimum of the fluctuations of the order parameter of seismicity, which is observed simultaneously with the initiation of an SES activity [50], appears when long range correlations prevail [51].

The scope of this paper is twofold. First, we report the geoelectrical field changes (SES) observed before the M${}_{w}$6.8 EQ that occurred on 25 October 2018. Second, we present the natural time analysis of both the SES activity and the seismicity preceding this EQ. The paper is structured as follows: the background of natural time analysis is presented in the next section. In the subsequent section, we give the results obtained. A discussion follows in Section 4 and a summary of our results and the main conclusions are presented in the final section.

## 2. Natural Time Analysis Background

Natural time analysis, introduced in the beginning of the 2000s [35,36,37,38,39], uncovers unique dynamic features hidden behind the time series of complex systems. In a time series comprising *N* events, the natural time $\chi_{k}=k/N$ serves as an index for the occurrence of the *k*-th event. This index together with the energy $Q_{k}$ released during the *k*-th event, i.e., the pair $\left(\chi_{k},Q_{k}\right)$, is studied in natural time analysis. Alternatively, one studies the pair $\left(\chi_{k},p_{k}\right)$, where

(1) $$p_{k}=\frac{Q_{k}}{\sum_{n=1}^{N}Q_{n}}$$

stands for the normalized energy released during the *k*-th event. As is obvious from Equation (1), the correct estimation of $p_{k}$ simply demands that $Q_{k}$ should be proportional to the energy emitted during the *k*-th event. Thus, for SES activities, $Q_{k}$ is proportional to the duration of the *k*-th pulse while, for EQs, it is proportional to the energy emitted [52] during the *k*-th EQ of magnitude $M_{k}$, i.e., $Q_{k}\propto 10^{1.5M_{k}}$ (see also [6,53]). The variance of χ weighted for $p_{k}$, labeled by $\kappa_{1}$, is given by [6,35,38,39,40]

(2) $$\kappa_{1}=\sum_{k=1}^{N}p_{k}{\left(\chi_{k}\right)}^{2}-{\left(\sum_{k=1}^{N}p_{k}\chi_{k}\right)}^{2}.$$

For the case of seismicity, the quantity $\kappa_{1}$ has been proposed to be an order parameter since $\kappa_{1}$ changes abruptly when a mainshock (the new phase) occurs, and, in addition, the statistical properties of its fluctuations are similar to those in other non-equilibrium and equilibrium critical systems ([40], see also pp. 249–253 of Reference [6]). It has been also found that $\kappa_{1}$ is a key parameter that enables recognition of the complex dynamical system under study entering the critical stage [6,35,36,37]. This occurs when $\kappa_{1}$ becomes equal to 0.070 {([54], see also page 343 of Reference [6]). In Table 8.1 of Reference [6], one can find a compilation of 14 cases including a variety of dynamical models in which the condition $\kappa_{1}$ = 0.070 has been ascertained (cf. this has been also later confirmed in the analyses of very recent experimental results in Japan by Hayakawa and coworkers [55,56,57]). Especially for the case of SES activities, it has been found that, when they are analyzed in natural time, we find $\kappa_{1}$ values close to 0.070 ([35,36,38,39], e.g., see Table 4.6 on p. 227 of Reference [6]), i.e.,

(3) $$\kappa_{1}\approx 0.070.$$

when analyzing in natural time the small EQs with magnitudes greater than or equal to a threshold magnitude $M_{thres}$ that occur after the initiation of an SES activity within the selectivity map of the measuring station that recorded the SES activity, the condition $\kappa_{1}=0.070$ is found to hold for a variety of $M_{thres}$ a few days up to one week before the strong EQ occurrence [6,30,40,41,44,45,46,47,54,58] . This is very important from a practical point of view because it enables the estimation of the occurrence time of a strong EQ with an accuracy of one week or so.

The entropy *S* in natural time is defined [6,35,39,53,59] by the relation

(4) $$S=\sum_{k=1}^{N}p_{k}\chi_{k}\ln\chi_{k}-\left(\sum_{k=1}^{N}p_{k}\chi_{k}\right)\ln\left(\sum_{m=1}^{N}p_{m}\chi_{m}\right).$$

It is a dynamic entropy showing [60] positivity, concavity and Lesche [61,62] experimental stability. When $Q_{k}$ are independent and identically-distributed random variables, *S* approaches [59] the value $S_{u}\equiv \frac{\ln2}{2}-\frac{1}{4}\approx 0.0966$ that corresponds to the case $Q_{k}=1/N$, which within the context of natural time is usually termed “uniform” distribution [6,39,53]. Notably, *S* changes its value to $S_{-}$ upon time-reversal, i.e., when the first event becomes last ($Q_{1}\to Q_{N}$), the second last but one ($Q_{2}\to Q_{N-1}$), etc.,

(5) $$S_{-}=\sum_{k=1}^{N}p_{N-k+1}\chi_{k}\ln\chi_{k}-\left(\sum_{k=1}^{N}p_{N-k+1}\chi_{k}\right)\ln\left(\sum_{m=1}^{N}p_{N-m+1}\chi_{m}\right),$$

and hence it gives us the possibility to observe the (true) time-arrow [60]. Interestingly, it has been established [6,53] that both *S* and $S_{-}$ for SES activities are smaller than $S_{u}$,

(6) $$S,S_{-}\leq S_{u}.$$

On the other hand, these conditions are violated for a variety of similar looking electrical noises (e.g., see Table 4.6 on p. 228 of Reference [6]).

Natural time has been recently employed by Turcotte and coworkers [63,64,65,66] as a basis for a new method to estimate the current level of seismic risk called “earthquake nowcasting”. This will be explained in the next section.

## 3. Results

### 3.1. Geoelectric Field Changes

Figure 2 depicts an SES activity that was recorded in the PIR station (see Figure 1), which comprises a multitude of measuring dipoles, on 2 October 2018 between 04:20 a.m. and 05:05 a.m. UTC. The potential differences ΔV of three of these electric dipoles of comparable length *L* (a few km) deployed in the NEE direction are shown. The true headings of these dipoles are from top to bottom in Figure 2 are 75.48${}^{\circ }$, 64.83${}^{\circ }$, and 76.16${}^{\circ }$ . An inspection of this figure reveals that the SES activity resembles a telegraph signal with periods of activity and periods of inactivity as it is usually the case [36,38,39]. If we impose a threshold in the ΔV variation [36,38,39], we can obtain the dichotomous (0–1) representation of the SES activity depicted by the cyan color in Figure 2.

### 3.2. Natural Time Analysis of Geoelectrical Signals. Criteria for Distinguishing SES

Apart from the aforementioned ΔV/L criterion suggested long ago for the distinction of SES from man-made noise [9], natural time analysis has provided, as mentioned, three additional criteria for the classification of an electric signal as SES activity. These criteria are Equation (3) and the conditions (6). The analysis in natural time of the dichotomous representation shown in Figure 2 results in $\kappa_{1}=0.072\left(2\right)$, S=0.066(2) and $S_{-}=0.079\left(3\right)$, which are obviously compatible with the criteria for distinguishing SES from noise. This leads us to support that the anomalous variation of the electric field of the Earth observed on 2 October 2018 is indeed an SES activity.

### 3.3. Estimation of the Occurrence Time of the Impending EQ

**Figure 3:**
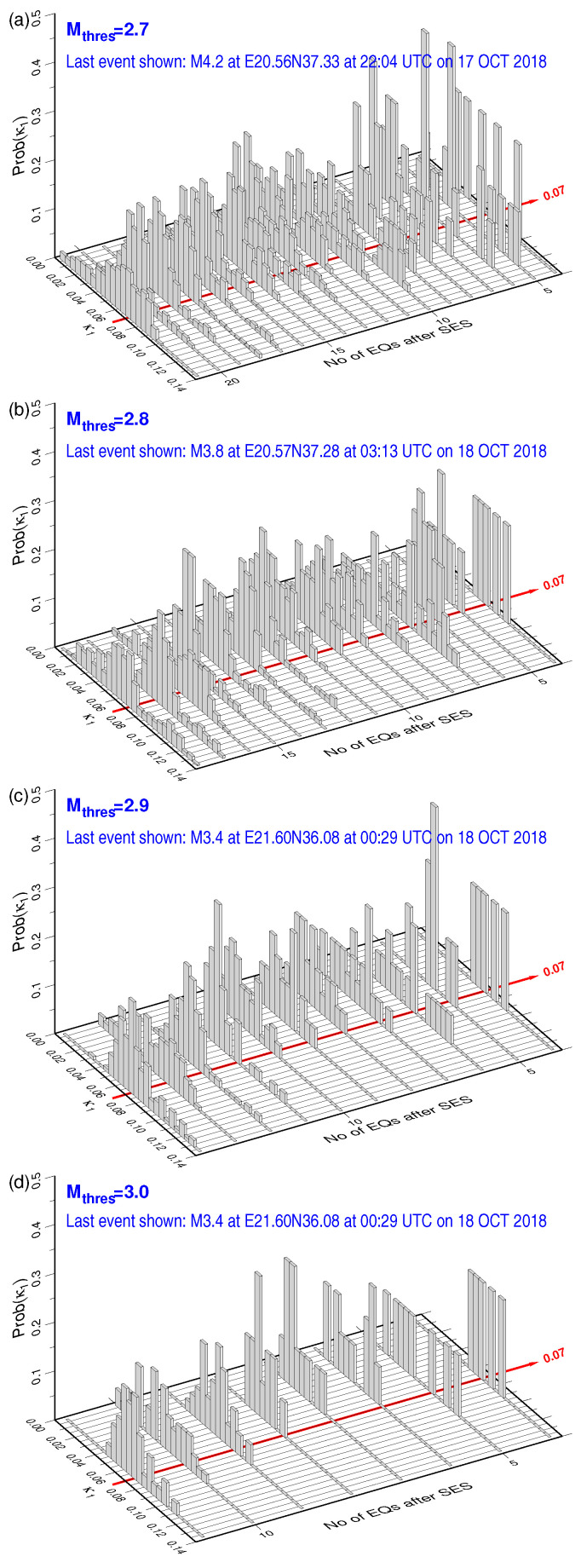
(**a**–**d**): the probability distribution Prob$\left(\kappa_{1}\right)$ of $\kappa_{1}$ versus $\kappa_{1}$ as it results after the occurrence of each small EQ within the selectivity area of PIR (see the gray shaded area in Figure 1) for various magnitude thresholds $M_{thres}$ = 2.7, 2.8, 2.9, and 3.0.

We now follow the method suggested in Reference [30] for the estimation of the occurrence time of the impending strong EQ by analyzing in natural time all the small EQs of magnitude greater than or equal to $M_{thres}$ that occurred after the initiation of the SES activity recorded on 2 October 2018 within the selectivity map of PIR measuring station shown by the gray shaded area in Figure 1. The EQ catalog [67] of the Institute of Geodynamics of the National Observatory of Athens has been used and, each time a new small EQ takes place, we calculate the $\kappa_{1}$ values corresponding to the events that occurred within all the possible subareas of the PIR selectivity map that include this EQ [30]. This procedure leads to an ensemble of $\kappa_{1}$ values from which we can calculate the probability Prob($\kappa_{1}$) of $\kappa_{1}$ to lie within $\kappa_{1}\pm 0.025$. Figure 3a–d depict the histograms of Prob($\kappa_{1}$) obtained after the occurrence of each small EQ with magnitude greater than or equal to 2.7, 2.8, 2.9, and 3.0, respectively. We observe that, within a period of 5 h around 18 October 2018 00:30 a.m. UTC, all four of the distributions Prob($\kappa_{1}$) exhibit a maximum at $\kappa_{1}=0.070$. This behavior has been found, as already mentioned, to occur a few days up to one week or so before the strong EQ occurrence [6,30,45,46,47]. Actually, one week later, i.e., on 25 October 2018, a strong M${}_{w}$6.8 EQ occurred [1] within the selectivity map of the PIR measuring station (see the red star in Figure 1). Interestingly, as it is written in the legends of the panels of Figure 3, two of the three small EQs that led to the fulfillment of the criticality condition $\kappa_{1}=0.070$ originated from epicentral areas located only 20 or 25 km south that of the strong EQ.

### 3.4. Estimation of the Current Level of Risk by Applying EQ Nowcasting

**Figure 4:**
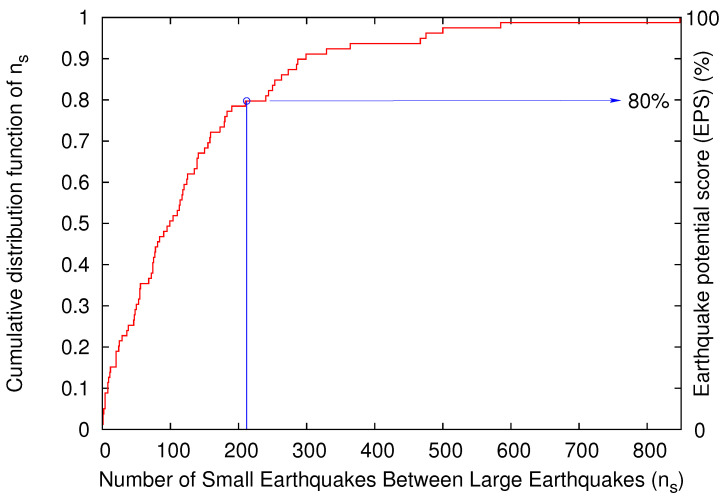
Estimation of the EQ potential score (EPS) based on the cumulative distribution function of the number $n_{s}$ of the small EQs (6.0>M≥4.0) that occur within the large area $N_{29}^{47}E_{12}^{35}$ depicted in Figure 1 between the occurrence of two strong (M≥6.0) EQs. The number ${\tilde{n}}_{s}$ of small EQs that occurred within R≤225 km with depths D≤200 km from the city of Patras since the M${}_{w}$6.5 EQ on 17 November 2015 07:11 a.m. UTC [74] and before the occurrence of the M${}_{w}$6.8 EQ on 25 October 2018 was 212.

Nowcasting EQs is a recent method for the determination of the current state of a fault system and the estimation of the current progress in the EQ cycle [63]. It uses a global EQ catalog to calculate from “small” EQs the level of hazard for “large” EQs. This is achieved by employing the natural time concept and counting the number $n_{s}$ of “small” EQs that occur after a “strong” EQ. The current value n(t) of $n_{s}$ since the occurrence of the last “strong” EQ is compared with the cumulative distribution function (cdf) $P(n_{s}<n\left(t\right))$ of $n_{s}$ obtained when ensuring that we have enough data to span at least 20 or more “large” EQ cycles. The EQ potential score (EPS) which equals the “current” cdf value, EPS = $P(n_{s}<n\left(t\right))$ is therefore a unique measure of the current level of hazard and assigns a number between 0% and 100% to every region so defined. Nowcasting EQs has already found many useful applications [64,65,66] among which is the estimation of seismic risk to Global Megacities. For this application [64], the EQs with depths smaller than a certain value *D* within a larger area are studied in order to obtain the cdf $P(n_{s}<n\left(t\right))$. Then, the number ${\tilde{n}}_{s}$ of “small” EQs around a Megacity, e.g., EQs in a circular region of epicentral distances smaller than a radius *R* with hypocenters shallower than *D*, is counted since the occurrence of the last “strong” EQ in this region. Based on the ergodicity of EQs that has been proven [68,69,70] by using the metric published in References [71,72], Rundle et al. [64] suggested that the seismic risk around a Megacity can be estimated by using the EPS corresponding to the current ${\tilde{n}}_{s}$ estimated in the circular region. Especially in their Figure 2, they used the large area $N_{29}^{47}E_{12}^{35}$ in order to estimate the EPS for EQs of magnitude greater than or equal to 6.5 at an area of radius *R* = 400 km around the capital of Athens in Greece. Figure 4 shows the results of a similar calculation based on the United States National EQ Information Center PDE catalog (the data of which are available from Reference [73]), which we performed focusing on the city of Patras, Greece, for EQs of magnitude greater than or equal to 6.0. Notably, before the occurrence of the M${}_{w}$6.8 EQ on 25 October 2018, EPS was found to be as high as 80%.

## 4. Discussion

Recently, the statistical significance of the Earth’s electric and magnetic field variations preceding EQs has been studied [75] on the basis of the modern tools of event coincidence analysis [76,77,78] and receiver operating characteristics [79,80]. Using an SES dataset [9,10,81] from the 1980s, it was found that SES are statistically significant precursors to EQs for lead times in the following four distinct time periods: 3 to 9 days, 18 to 24 days, 43 to 47 days, and 58 to 62 days (the first one corresponds to single SES, while the latter to three SES activities [75]). Since the SES activity, shown in Figure 2, was recorded on 2 October 2018, the SES lead time for the present case of the M${}_{w}$6.8 EQ on 25 October 2018, which is 23 days, falls favorably within the second time period of 18 to 24 days. Moreover, the analysis of the seismicity subsequent to the initiation of the SES activity in the selectivity area of the PIR station has led to the conclusion that the criticality condition $\kappa_{1}=0.070$ has been satisfied early in the morning on 18 October 2018. This compares favorably with the time window of a few days up to one week already found from various SES activities in Greece, Japan and United States [6,30,45,46,47,58].

Let us now turn to the results concerning the entropy of the SES activity of Figure 2 in natural time. As it was reported, both *S* and $S_{-}$ are well below $S_{u}$ in accordance with the findings (e.g., see Reference [53]) so far for SES activities. Based on the critical properties that characterize the emission of signals that precede rupture (i.e., infinite range correlations compatible with a detrended fluctuation analysis (DFA) [82,83,84] exponent $\alpha_{DFA}=1$), a fractional Brownian motion [85,86] model has been suggested [41] according to which both *S* and $S_{-}$ values should scatter around 0.079 with a standard deviation of 0.011 (see Figure 4 of Reference [41]). Interestingly, the values S=0.066(2) and $S_{-}=0.079\left(3\right)$ of the SES activity recorded on 2 October 2018 are fully compatible with this model.

Finally, the successful results (i.e., the 80% EPS found before the occurrence of the M${}_{w}$6.8 EQ on 25 October 2018) from the EQ nowcasting method, which is based on natural time, are very promising. Nowcasting does not involve any model and there are no free parameters to be fit to the data [63].

## 5. Conclusions

The strong EQ of magnitude M${}_{w}$6.8 that occurred in Western Greece on 25 October 2018 was preceded by an SES activity on 2 October 2018 recorded at the PIR measuring station of the VAN telemetric network. The EQ epicenter was located within the selectivity map of PIR depicted by the gray shaded area in Figure 1.

The lead time of 23 days between the precursory SES activity and the strong EQ is statistically significant as recently found by the recent methods of event coincidence analysis and receiver operating characteristics. Both the entropy *S* and the entropy $S_{-}$ under time reversal in natural time are compatible with previous observation for SES activities as well as agree with a model for SES activities based on fractional Brownian motion. The analysis in natural time of the seismicity subsequent to the SES activity by considering the events occurring within the selectivity area of PIR shows that criticality has been reached early in the morning on 18 October 2018, almost a week before the strong EQ occurrence, in accordance with the earlier findings. Finally, EQ nowcasting has revealed an 80% EPS. In general, here we showed that the strong EQ under discussion provides an excellent validation of the methods developed so far in natural time for EQ prediction.

## References

[1] United States Geological Survey, Earthquake Hazards Program M6.8-33km SW of Mouzaki, Greece. https://earthquake.usgs.gov/earthquakes/eventpage/us1000hhb1/technical.

[2] United States Geological Survey, Earthquake Hazards Program M5.0-30km SSW of Lithakia, Greece. https://earthquake.usgs.gov/earthquakes/eventpage/us1000hhay/technical.

[3] Uyeda S., Al-Damegh E., Dologlou E., Nagao T. (1999). Some relationship between VAN seismic electric signals (SES) and earthquake parameters. Tectonophysics.

[4] Varotsos P., Eftaxias K., Lazaridou M., Nomicos K., Sarlis N., Bogris N., Makris J., Antonopoulos G., Kopanas J. (1996). Recent earthquake prediction results in Greece based on the observation of Seismic Electric Signals. Acta Geophys. Pol..

[5] Varotsos P. (2005). The Physics of Seismic Electric Signals.

[6] Varotsos P.A., Sarlis N.V., Skordas E.S. (2011). Natural Time Analysis: The New View of Time. Precursory Seismic Electric Signals, Earthquakes and Other Complex Time-Series.

[7] Varotsos P., Alexopoulos K. (1984). Physical Properties of the variations of the electric field of the earth preceding earthquakes, I. Tectonophysics.

[8] Varotsos P., Alexopoulos K. (1984). Physical Properties of the variations of the electric field of the earth preceding earthquakes, II. Tectonophysics.

[9] Varotsos P., Lazaridou M. (1991). Latest aspects of earthquake prediction in Greece based on Seismic Electric Signals. Tectonophysics.

[10] Varotsos P., Alexopoulos K., Lazaridou M. (1993). Latest aspects of earthquake prediction in Greece based on Seismic Electric Signals, II. Tectonophysics.

[11] Varotsos P.A., Sarlis N.V., Skordas E.S., Lazaridou M.S. (2008). Fluctuations, under time reversal, of the natural time and the entropy distinguish similar looking electric signals of different dynamics. J. Appl. Phys..

[12] Uyeda S., Nagao T., Orihara Y., Yamaguchi T., Takahashi I. (2000). Geoelectric potential changes: Possible precursors to earthquakes in Japan. Proc. Natl. Acad. Sci. USA.

[13] Uyeda S., Hayakawa M., Nagao T., Molchanov O., Hattori K., Orihara Y., Gotoh K., Akinaga Y., Tanaka H. (2002). Electric and magnetic phenomena observed before the volcano-seismic activity in 2000 in the Izu Island Region, Japan. Proc. Natl. Acad. Sci. USA.

[14] Orihara Y., Kamogawa M., Nagao T., Uyeda S. (2012). Preseismic anomalous telluric current signals observed in Kozu-shima Island, Japan. Proc. Natl. Acad. Sci. USA.

[15] Zlotnicki J., Kossobokov V., Le Mouël J.L. (2001). Frequency spectral properties of an ULF electromagnetic signal around the 21 July 1995, M = 5.7, Yong Deng (China) earthquake. Tectonophysics.

[16] Huang Q. (2011). Retrospective investigation of geophysical data possibly associated with the Ms8.0 Wenchuan earthquake in Sichuan, China. J. Asian Earth Sci..

[17] Gao S.D., Tang J., Du X.B., Liu X.F., Su Y.G., Chen Y.P., Di G.R., Mei D.L., Zhan Y., Wang L.F. (2010). The change characteristics of electromagnetic field before to after Wenchuan M_s_8.0 earthquake. Chin. J. Geophys..

[18] Fan Y.Y., Du X.B., Zlotnicki J., Tan D.C., An Z.H., Chen J.Y., Zheng G.L., Liu J., Xie T. (2010). The Electromagnetic Phenomena Before the Ms8.0 Wenchuan Earthquake. Chin. J. Geophys..

[19] Ramírez-Rojas A., Flores-Márquez E.L., Guzmán-Vargas L., Gálvez-Coyt G., Telesca L., Angulo-Brown F. (2008). Statistical features of seismoelectric signals prior to M7.4 Guerrero-Oaxaca earthquake (México). Nat. Hazards Earth Syst. Sci..

[20] Ramírez-Rojas A., Telesca L., Angulo-Brown F. (2011). Entropy of geoelectrical time series in the natural time domain. Nat. Hazards Earth Syst. Sci..

[21] Sarlis N.V., Varotsos P.A., Skordas E.S., Zlotnicki J., Nagao T., Rybin A., Lazaridou-Varotsos M.S., Papadopoulou K. (2018). Seismic electric signals in seismic prone areas. Earthq. Sci..

[22] Varotsos P., Alexopoulos K. (1986). Thermodynamics of Point Defects and Their Relation with Bulk Properties.

[23] Varotsos P., Miliotis D. (1974). New aspects on the dielectric properties of the alkali halides with divalent impurities. J. Phys. Chem. Solids.

[24] Lazaridou M., Varotsos C., Alexopoulos K., Varotsos P. (1985). Point-defect parameters of LiF. J. Phys. C Solid State.

[25] Varotsos P. (2008). Point defect parameters in *β*-PbF_2_ revisited. Solid State Ion..

[26] Varotsos P., Alexopoulos K. (1987). Physical properties of the variations in the electric field of the earth preceding earthquakes, III. Tectonophysics.

[27] Varotsos P., Lazaridou M., Eftaxias K., Antonopoulos G., Makris J., Kopanas J., Lighthill S.J. (1996). Short term earthquake prediction in Greece by Seismic Electric Signals. The Critical Review of VAN: Earthquake Prediction from Seismic Electric Signals.

[28] Lazaridou-Varotsos M.S. (2013). Earthquake Prediction by Seismic Electric Signals: The Success of the VAN Method over Thirty Years.

[29] Uyeda S., Lighthill S.J. (1996). Introduction to the VAN method of earthquake prediction. The Critical Review of VAN: Earthquake Prediction from Seismic Electric Signals.

[30] Sarlis N.V., Skordas E.S., Lazaridou M.S., Varotsos P.A. (2008). Investigation of seismicity after the initiation of a Seismic Electric Signal activity until the main shock. Proc. Jpn. Acad. Ser. B Phys. Biol. Sci..

[31] Varotsos P., Sarlis N., Lazaridou M., Kapiris P. (1998). Transmission of stress induced electric signals in dielectric media. J. Appl. Phys..

[32] Sarlis N., Lazaridou M., Kapiris P., Varotsos P. (1999). Numerical Model of the Selectivity Effect and ΔV/L criterion. Geophys. Res. Lett..

[33] Varotsos P., Sarlis N., Lazaridou M. (2000). Transmission of stress induced electric signals in dielectric media. Part II. Acta Geophys. Pol..

[34] Varotsos P., Sarlis N., Skordas E. (2000). Transmission of stress induced electric signals in dielectric media. Part III. Acta Geophys. Pol..

[35] Varotsos P.A., Sarlis N.V., Skordas E.S. (2001). Spatio-Temporal complexity aspects on the interrelation between Seismic Electric Signals and Seismicity. Pract. Athens Acad..

[36] Varotsos P.A., Sarlis N.V., Skordas E.S. (2002). Long-range correlations in the electric signals that precede rupture. Phys. Rev. E.

[37] Varotsos P.A., Sarlis N.V., Skordas E.S. (2002). Seismic Electric Signals and Seismicity: On a tentative interrelation between their spectral content. Acta Geophys. Pol..

[38] Varotsos P.A., Sarlis N.V., Skordas E.S. (2003). Long-range correlations in the electric signals the precede rupture: Further investigations. Phys. Rev. E.

[39] Varotsos P.A., Sarlis N.V., Skordas E.S. (2003). Attempt to distinguish electric signals of a dichotomous nature. Phys. Rev. E.

[40] Varotsos P.A., Sarlis N.V., Tanaka H.K., Skordas E.S. (2005). Similarity of fluctuations in correlated systems: The case of seismicity. Phys. Rev. E.

[41] Varotsos P.A., Sarlis N.V., Skordas E.S., Tanaka H.K., Lazaridou M.S. (2006). Entropy of seismic electric signals: Analysis in the natural time under time reversal. Phys. Rev. E.

[42] Varotsos P.A., Sarlis N.V., Skordas E.S., Tanaka H.K., Lazaridou M.S. (2006). Attempt to distinguish long-range temporal correlations from the statistics of the increments by natural time analysis. Phys. Rev. E.

[43] Varotsos P.A., Sarlis N.V., Skordas E.S. (2009). Detrended fluctuation analysis of the magnetic and electric field variations that precede rupture. Chaos.

[44] Varotsos P.A., Sarlis N.V., Skordas E.S., Lazaridou M.S. (2007). Identifying sudden cardiac death risk and specifying its occurrence time by analyzing electrocardiograms in natural time. Appl. Phys. Lett..

[45] Varotsos P.A., Sarlis N.V., Skordas E.S., Uyeda S., Kamogawa M. (2010). Natural time analysis of critical phenomena. The case of Seismicity. EPL.

[46] Varotsos P.A., Sarlis N.V., Skordas E.S., Christopoulos S.R.G., Lazaridou-Varotsos M.S. (2015). Identifying the occurrence time of an impending mainshock: A very recent case. Earthq. Sci..

[47] Varotsos P.A., Sarlis N.V., Skordas E.S. (2017). Identifying the occurrence time of an impending major earthquake: A review. Earthq. Sci..

[48] Sarlis N.V., Skordas E.S., Varotsos P.A., Nagao T., Kamogawa M., Tanaka H., Uyeda S. (2013). Minimum of the order parameter fluctuations of seismicity before major earthquakes in Japan. Proc. Natl. Acad. Sci. USA.

[49] Sarlis N.V., Skordas E.S., Varotsos P.A., Nagao T., Kamogawa M., Uyeda S. (2015). Spatiotemporal variations of seismicity before major earthquakes in the Japanese area and their relation with the epicentral locations. Proc. Natl. Acad. Sci. USA.

[50] Varotsos P.A., Sarlis N.V., Skordas E.S., Lazaridou M.S. (2013). Seismic Electric Signals: An additional fact showing their physical interconnection with seismicity. Tectonophysics.

[51] Varotsos P.A., Sarlis N.V., Skordas E.S. (2014). Study of the temporal correlations in the magnitude time series before major earthquakes in Japan. J. Geophys. Res. Space Phys..

[52] Kanamori H. (1978). Quantification of Earthquakes. Nature.

[53] Sarlis N.V. (2017). Entropy in Natural Time and the Associated Complexity Measures. Entropy.

[54] Varotsos P., Sarlis N.V., Skordas E.S., Uyeda S., Kamogawa M. (2011). Natural time analysis of critical phenomena. Proc. Natl. Acad. Sci. USA.

[55] Hayakawa M., Schekotov A., Potirakis S., Eftaxias K. (2015). Criticality features in ULF magnetic fields prior to the 2011 Tohoku earthquake. Proc. Jpn Acad. Ser. B Phys. Biol. Sci..

[56] Potirakis S.M., Asano T., Hayakawa M. (2018). Criticality Analysis of the Lower Ionosphere Perturbations Prior to the 2016 Kumamoto (Japan) Earthquakes as Based on VLF Electromagnetic Wave Propagation Data Observed at Multiple Stations. Entropy.

[57] Potirakis S.M., Schekotov A., Asano T., Hayakawa M. (2018). Natural time analysis on the ultra-low frequency magnetic field variations prior to the 2016 Kumamoto (Japan) earthquakes. J. Asian Earth Sci..

[58] Uyeda S., Kamogawa M., Tanaka H. (2009). Analysis of electrical activity and seismicity in the natural time domain for the volcanic-seismic swarm activity in 2000 in the Izu Island region, Japan. J. Geophys. Res..

[59] Varotsos P.A., Sarlis N.V., Skordas E.S., Lazaridou M.S. (2004). Entropy in Natural Time Domain. Phys. Rev. E.

[60] Varotsos P.A., Sarlis N.V., Tanaka H.K., Skordas E.S. (2005). Some properties of the entropy in the natural time. Phys. Rev. E.

[61] Lesche B. (1982). Instabilities of Renyi entropies. J. Stat. Phys..

[62] Lesche B. (2004). Renyi entropies and observables. Phys. Rev. E.

[63] Rundle J.B., Turcotte D.L., Donnellan A., Grant Ludwig L., Luginbuhl M., Gong G. (2016). Nowcasting earthquakes. Earth Space Sci..

[64] Rundle J.B., Luginbuhl M., Giguere A., Turcotte D.L. (2018). Natural Time, Nowcasting and the Physics of Earthquakes: Estimation of Seismic Risk to Global Megacities. Pure Appl. Geophys..

[65] Luginbuhl M., Rundle J.B., Hawkins A., Turcotte D.L. (2018). Nowcasting Earthquakes: A Comparison of Induced Earthquakes in Oklahoma and at the Geysers, California. Pure Appl. Geophys..

[66] Luginbuhl M., Rundle J.B., Turcotte D.L. (2018). Natural Time and Nowcasting Earthquakes: Are Large Global Earthquakes Temporally Clustered?. Pure Appl. Geophys..

[67] National Observatory of Athens, Institute of Geodynamics Recent Earthquakes. http://www.gein.noa.gr/en/seismicity/recent-earthquakes.

[68] Ferguson C.D., Klein W., Rundle J.B. (1999). Spinodals, scaling, and ergodicity in a threshold model with long-range stress transfer. Phys. Rev. E.

[69] Tiampo K.F., Rundle J.B., Klein W., Martins J.S.S., Ferguson C.D. (2003). Ergodic Dynamics in a Natural Threshold System. Phys. Rev. Lett..

[70] Tiampo K.F., Rundle J.B., Klein W., Holliday J., Sá Martins J.S., Ferguson C.D. (2007). Ergodicity in natural earthquake fault networks. Phys. Rev. E.

[71] Thirumalai D., Mountain R.D., Kirkpatrick T.R. (1989). Ergodic behavior in supercooled liquids and in glasses. Phys. Rev. A.

[72] Mountain R.D., Thirumalai D. (1992). Ergodicity and activated dynamics in supercooled liquids. Phys. Rev. A.

[73] United States Geological Survey, Earthquake Hazards Program Search Earthquake Catalog. https://earthquake.usgs.gov/earthquakes/eventpage/us1000hhb1/technical.

[74] United States Geological Survey, Earthquake Hazards Program M6.5-10km WSW of Nidri, Greece. https://earthquake.usgs.gov/earthquakes/eventpage/us10003ywp/technical.

[75] Sarlis N.V. (2018). Statistical Significance of Earth’s Electric and Magnetic Field Variations Preceding Earthquakes in Greece and Japan Revisited. Entropy.

[76] Donges J., Schleussner C.F., Siegmund J., Donner R. (2016). Event coincidence analysis for quantifying statistical interrelationships between event time series. Eur. Phys. J. Spec. Top..

[77] Schleussner C.F., Donges J.F., Donner R.V., Schellnhuber H.J. (2016). Armed-conflict risks enhanced by climate-related disasters in ethnically fractionalized countries. Proc. Natl. Acad. Sci. USA.

[78] Siegmund J.F., Siegmund N., Donner R.V. (2017). CoinCalc—A new R package for quantifying simultaneities of event series. Comput. Geosci..

[79] Fawcett T. (2006). An introduction to ROC analysis. Pattern Recogn. Lett..

[80] Sarlis N.V., Christopoulos S.R.G. (2014). Visualization of the significance of Receiver Operating Characteristics based on confidence ellipses. Comput. Phys. Commun..

[81] Dologlou E. (1993). A three year continuous sample of officially documented predictions issued in Greece using the VAN method: 1987–1989. Tectonophysics.

[82] Peng C.K., Buldyrev S.V., Havlin S., Simons M., Stanley H.E., Goldberger A.L. (1994). Mosaic organization of DNA nucleotides. Phys. Rev. E.

[83] Peng C.K., Buldyrev S.V., Goldberger A.L., Havlin S., Mantegna R.N., Simons M., Stanley H.E. (1995). Statistical properties of DNA sequences. Phys. A.

[84] Kantelhardt J.W., Koscielny-Bunde E., Rego H.H.A., Havlin S., Bunde A. (2001). Detecting long-range correlations with detrended fluctuation analysis. Phys. A.

[85] Mandelbrot B.B., van Ness J.W. (1968). Fractional Noises and Applications. SIAM Rev..

[86] Mandelbrot B.B., Wallis J.R. (1969). Some long-run properties of geophysical records. Water Resour. Res..

